# Screening of Phytotoxins in Raw Honey and the Honey Sugar Matrix’s Modulatory Effects on Their Toxicity

**DOI:** 10.3390/foods15061058

**Published:** 2026-03-17

**Authors:** Liuqing Yang, Tian Xiao, Xin Yang, Li Yang, Wenjing Shen, Zihao Huang, Guang Nie, Conghui Dong, Xiue Jin, Qi Tang, Ying Lu, Yajie Zheng

**Affiliations:** 1 National Research Center of Engineering and Technology for Utilization of Botanical Functional Ingredients, Changsha 410128, China; yalq121@163.com (L.Y.); qqd260817@163.com (W.S.); 18567269782@163.com (C.D.); 2 College of Horticulture, Hunan Agricultural University, Changsha 410128, China; xiao991105@163.com (T.X.); lyy1203438808@126.com (L.Y.); nnra3q@163.com (Z.H.); ng2597568576@163.com (G.N.); tangqi@hunau.edu.cn (Q.T.); 3 Ganzhou Forestry Science Research Institute, Ganzhou 341000, China; lp0706091999@126.com; 4 Hubei Institute of Veterinary Drug Control, Wuhan 430000, China; m13797009870@163.com; 5 Yuelushan Laboratory, Changsha 410128, China

**Keywords:** honey, phytotoxins, matrix effect, food safety, LC-MS/MS

## Abstract

Honey, as a natural and nutritious sweetener, is one of the most widely consumed foods worldwide. However, the presence of phytotoxins in honey and the influence of honey’s intrinsic sugar matrix on the toxicity of these phytotoxins remain insufficiently explored. An optimized liquid chromatography–quadrupole trap tandem mass spectrometry method was developed to quantify 17 toxic alkaloids in 150 raw honey samples. Camptothecin was identified for the first time in the tested samples and was the most prevalent contaminant (36% detection, max 3.09 μg/kg), which induced cardiac hypertrophy and impaired cardiac function in zebrafish assays. The honey sugar matrix further potentiated these adverse cardiac effects through exacerbating oxidative stress and upregulating pro-inflammatory and pro-apoptotic gene expression, while natural honey partially mitigated such damage by upregulating the key antioxidant gene *nrf2*, thereby downregulating *il-1β* and regulating the *bcl2*/*bax* expression ratio. This study offers novel insights into honey phytotoxins’ matrix-modulated toxicity, laying a scientific foundation for optimizing safety protocols and matrix-specific risk standards.

## 1. Introduction

Honey is a natural sweetener produced by honeybees and contains various bioactive compounds including polyphenols, glycoproteins, and glycopeptides. These components underpin its longstanding use in traditional medicine for anti-inflammatory and detoxifying purposes [1]. Although these secondary metabolites provide significant therapeutic benefits, they can also introduce toxicological risks if phytotoxins are present within the honey [2]. Compared to filtered and diluted commercial honey, unprocessed raw honey carries more significant food safety risks due to its higher retention of phytotoxins [3]. Consequently, raw honey has been linked to severe poisoning incidents worldwide [4,5]. Among these, the well-known “mad honey disease” is primarily caused by grayanotoxins found in the nectar of plants from the Ericaceae family [5]. These toxins induce sustained depolarization of nerve and cardiac muscle cells, leading to severe cardiac and neurotoxic symptoms such as sinus bradycardia, hypotension, and loss of consciousness [6]. As global interest in exotic natural products grows, intoxications associated with “mad honey” are no longer confined to their traditional origins in Turkey and the Himalayas [3]. The geographical expansion of these cases highlights the necessity of establishing rigorous screening methods for phytotoxin components in honey.

Recent studies have shown that the regulatory effect of sugars on bioavailability can significantly alter the absorption characteristics of plant-derived compounds. The honey sugar matrix is defined as the concentrated aqueous environment primarily composed of fructose, glucose, and sucrose in specific proportions. In addition to analyzing individual toxins, the potential impact of this sugar matrix on overall toxicity must also be considered. The literature suggests that honey can function as a natural deep eutectic solvent (NADES), which is utilized in traditional Chinese medicine processing to enhance the solubility and oral bioavailability of bioactive compounds [7]. Furthermore, fructose-based systems have been reported to enhance certain biological activities, such as polyphenol-mediated inhibition. However, certain sugars may also potentiate the toxicity of specific plant-derived compounds like triptolide and celastrol. This dual effect suggests that the sugar matrix might exert a synergistic effect, and verifying this could lead to a critical refinement of safety evaluation standards for toxic honey.

To address this critical knowledge gap, this study aims to screen for 17 target phytotoxins in raw honey and validate the cardiotoxicity of identified toxins using an established zebrafish model. Crucially, Bliss independence modeling and mechanistic assays targeting oxidative stress, apoptosis, and inflammation are employed to investigate the interactive effects of the honey sugar matrix with these phytotoxins. This work is expected to provide experimental evidence for sugar–phytotoxin synergistic interactions, thereby advocating for a shift in risk assessment toward matrix-dependent toxicity evaluations.

## 2. Materials and Methods

### 2.1. Sample Collection and Preparation

A total of 150 raw honey samples were collected from Hubei, Hunan, and Guizhou provinces, China. Among these, 18 samples were harvested near *Macleaya cordata* and *Tripterygium wilfordii* plantations, while others were multifloral (Supplementary Materials Table S1). Commercial honey was bought from Beijing Tong Ren Tang (Changsha, China). Mimetic honey was prepared by dissolving fructose, glucose, and sucrose (12:10:1, *w*/*w*/*w*) in water to match the commercial honey’s sugar profile.

The QuEChERS procedure used in this study to perform the pretreatment of raw honey samples was detailed in our prior work [8].

### 2.2. LC-MS/MS Analysis

Targeted screening and quantitation were performed using a Shimadzu UFLC system (CBM-30A controller, Kyoto, Japan) coupled with an AB SCIEX 4500 QTRAP mass spectrometer (Applied Biosystems, Foster City, CA, USA). Chromatographic separation was achieved on a Waters ACQUITY UPLC HSS T3 column (100 × 2.1 mm, 1.8 μm, Waters, Milford, MA, USA) at 40 °C, using a 17 min gradient elution of 0.1% formic acid in water (A) and acetonitrile (B) at a flow rate of 0.35 mL/min. The mass spectrometer utilized an electrospray ionization (ESI) source in positive mode with multiple reaction monitoring (MRM) based on previously established protocols. Key parameters included an ion source voltage of 5500 V and temperature of 550 °C, with optimized declustering potentials (DP) and collision energies (CE) for all 17 analytes provided in Table S2.

### 2.3. Method Performance and Quantitation

The matrix effects (MEs) of 17 analytes were measured by diluting a mixed standard solution (2 μg/mL) to a final concentration of 10 ng/mL and 1 ng/mL in methanol and a mimetic honey matrix, respectively. The matrix factor for each analyte was calculated by ascertaining the ratio of the peak area in the presence of the matrix to that in methanol. The linearity was examined via sequential spiking of the mixed standard solution (2 μg/mL) into matrix extracts of mimetic honey as a diluent to achieve a concentration range of 0.01~500 ng/mL. The limits of quantitation (LOQs) were determined using the signal-to-noise (S/N) approach, with an S/N ratio greater than 10 for the LOQs. Precision was measured by six consecutive injections of a 20 ng/mL standard solution, repeatability was assessed from six parallel determinations, and stability was evaluated by detections at 0 h, 4 h, 8 h, 12 h, 16 h, 20 h, and 24 h, all of these evaluations were quantified using the relative standard deviations (RSDs) of the peak areas of these analytes. Recoveries were evaluated through the external standard method by spiking three distinct concentrations (low, medium, high) of the mixed standard solutions into five replicates of the mimetic honey samples, followed by the calculation based on measured and theoretical concentrations of spiked mixed standards.

Following validation, potential plant-derived components in raw honey samples were determined using the above LC-MS/MS method.

### 2.4. Animal Husbandry and Embryo Collection

Wild-type AB strain zebrafish (*Danio rerio*) were obtained from the China Zebrafish Resource Center (Wuhan, China). Adult zebrafish were acclimated under a 14 h light/10 h dark cycle in a recirculating system with dechlorinated culture water (pH 7.0–7.5; conductivity 500 ± 50 μS/cm; 27 ± 0.5 °C). For spawning, adult male and female fish (2:3 ratio) were paired overnight in spawning boxes, with light-induced mating. Eggs were collected the following morning and washed with culture water.

### 2.5. Test Solution Preparation for Acute Toxicity Tests

#### 2.5.1. Individual Compound Solutions

Stock solutions of protopine (40 mg/mL in DMSO), allocryptopine (40 mg/mL in DMSO), berberine (40 mg/mL in DMSO), veratramine (40 mg/mL in DMSO), and camptothecin (CPT, 4 mg/mL in DMSO) were prepared. Each stock solution was then serially diluted with culture water to generate working solutions at various concentrations as detailed in Table S3.

#### 2.5.2. Compound-Sugar Matrix Solutions

The CPT stock solution was diluted with culture water, as well as with culture water containing sugars (commercial honey, mimetic honey, glucose, or fructose) pre-diluted 120-fold. Commercial honey was included as a natural matrix control to evaluate the baseline influence of a complex biological sugar environment on CPT toxicity. This dilution factor (120-fold) was previously proven to be non-toxic in prior studies [9]. The dilution steps generated working solutions at various concentrations; detailed information is presented in Table S3.

### 2.6. Test Solution Exposure

Healthy three-day post-fertilization (3 dpf) zebrafish larvae were randomly distributed into 12-well plates (*n* = 20/well). Zebrafish larvae acute toxicity tests were conducted, with 0.1% DMSO solution as the solvent control group (SC) for comparison. The initial culture medium was removed, and then each well was filled with 3 mL of the corresponding working solution, with three replicates per concentration.

### 2.7. Morphological Examination

Following sample treatment, morphological examinations were performed under a stereomicroscope (Leica M205 FA, Wetzlar, Germany) to capture images. The median lethal concentration (LC_50_) was determined via probit analysis using GraphPad Prism 8.0.2.

### 2.8. Histopathological Examination

Following the acute toxicity tests, histopathological examinations were performed. The larvae were fixed in 4% paraformaldehyde, embedded in paraffin, and sectioned at 5 µm. Sections were stained with hematoxylin and eosin (H&E) and subsequently imaged using a NanoZoomer S360 digital slide scanner (HAMAMATSU PHOTONICS, Hamamatsu, Japan).

### 2.9. Cardiac Morpho-Functional Examination

Zebrafish larvae at 3 dpf were divided into four groups and exposed to the SC, CPT, CPT with mimetic honey (CPTM), or CPT with honey (CPTH). The concentration of all CPT-containing treatment groups was 0.05 μg/mL (approximate LC_10_ value of CPT). After 48 h of treatment exposure, larvae were imaged under the bright field using the fluorescence microscope. In each experiment, six zebrafish larvae were pooled together, and all experiments were replicated at least three times for reliability.

Cardiac parameters, including heart rate (HR), stroke volume (SV), ejection fraction (EF), and cardiac output (CO), were assessed in 5 dpf larvae following a reported method [9]. Larvae were recorded at 60 fps, and ventricular volumes were calculated by measuring the long and short axes using ImageJ software version 1.54n, assuming a prolate spheroidal heart shape.

### 2.10. Measurement of Oxidative Stress

A total of ten larvae at 5 dpf were stained with 5 µM DCFH-DA for 30 min and imaged via fluorescence microscopy. Fluorescence intensity was quantified using ImageJ and expressed relative to the control [10]. For biochemical analysis, 40 larvae were homogenized in 0.9% NaCl. Superoxide dismutase (SOD) and catalase (CAT) activities, as well as glutathione (GSH) and malondialdehyde (MDA) levels, were determined using kits from Nanjing Jiancheng Bioengineering Institute (Nanjing, China) according to the manufacturer’s protocols.

### 2.11. Apoptosis Assay

After 48 h treatment exposure, larvae were stained with 5 mg/L acridine orange (AO, Shanghai Macklin Biochemical Technology Co., Ltd., Shanghai, China) in the dark for 30 min, and then washed with culture water 3 times. The larvae were imaged using the fluorescence stereomicroscope as described above [11].

### 2.12. Gene Transcription Analysis

Thirty 5 dpf zebrafish larvae per replicate were collected. Total RNA was extracted using the TRIzol reagent (Hunan Acres Bioengineering Co., Ltd., Changsha, China) following the manufacturer’s instructions [12]. Then approximately 1 μg of total RNA was reverse transcribed to cDNA using the Evo M-MLV RT Mix kit (Acres). Real-time PCR (CFX96, Bio-Rad, Hercules, CA, USA) was performed in a total volume of 20 μL reactions containing cDNA, SYBR Green mixture, and gene-specific primer (Table S4). The gene expression level was quantified using the 2^−∆∆CT^ method and normalized to the *β-actin* gene.

### 2.13. Statistical Analyses

Data are expressed as mean ± SEM and analyzed using GraphPad Prism 8.0.2. Normality was assessed by Shapiro–Wilk test. Significant differences were determined via one-way ANOVA with Tukey’s post hoc test (*p* < 0.05). Synergism between CPT and honey sugar matrix was evaluated using Bliss independence modeling.

## 3. Results

### 3.1. Method Performance Evaluation

The analytical method was validated for sensitivity, linearity, matrix effects (MEs), precision, and recovery (Tables S5 and S6). Limits of quantitation (LOQs) ranged from 0.01 to 2.00 μg/kg. All analytes showed excellent linearity (*R*^2^ > 0.998) within their respective ranges. MEs (0.80–1.45) indicated minimal honey matrix impact on ionization. Precision (RSD < 13%), repeatability, and stability (RSD < 12%) were all within acceptable limits. Average recoveries at low, medium, and high spiked levels ranged from 62.48% to 124.50%, with RSDs generally below 12.5%, demonstrating the method’s reliability for honey analysis.

### 3.2. Quantitative Analysis of Target Compound in Raw Honey

**Figure 1:**
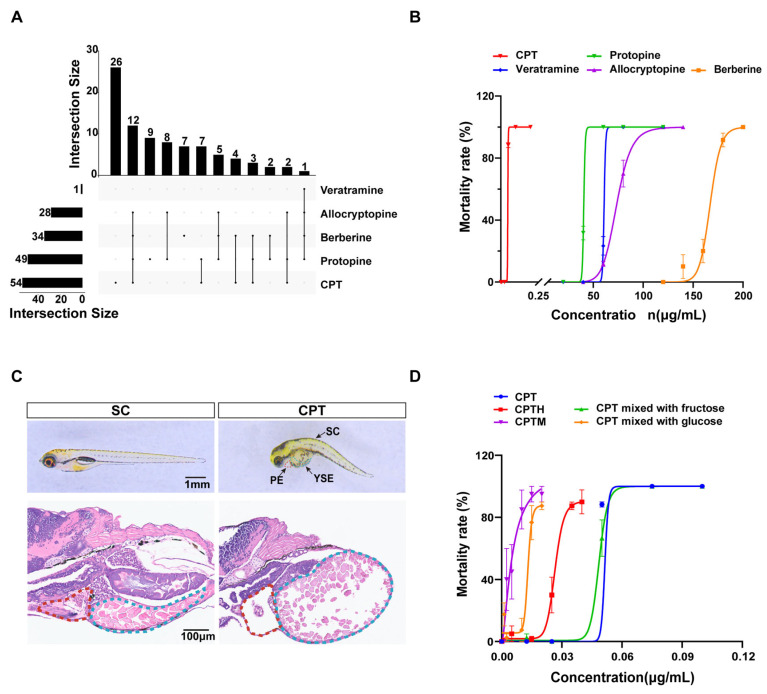
Phytotoxin identification in raw honey and acute toxicity assessment in zebrafish larvae (**A**) UpSet Venn diagram of differentially distributed phytotoxins identified by LC-MS/MS in raw honey. (**B**) Mortality curves in zebrafish larvae exposed to identified phytotoxins. (**C**) Morphological and histological phenotypes of zebrafish larvae after 48 h exposure to 0.05 μg·mL^−1^ CPT. (**D**) Mortality curves of zebrafish larvae co-exposure of CPT with honey sugar matrix. Note: PE, pericardial edema; YSE, yolk sac edema; CPT, camptothecin; CPTM, CPT with mimetic honey; CPTH, CPT with honey. Pericardial area and yolk sac are outlined in red and light blue, respectively.

LC-MS/MS analysis identified 5 of 17 target analytes in raw honey samples (Table S7). CPT was the most prevalent (36.0%, up to 3.09 μg/kg), followed by protopine (32.7%, 0.003–2.24 μg/kg), berberine (22.7%, up to 25.40 μg/kg), and allocryptopine (up to 2.00 μg/kg). Veratramine was detected in only one sample (1.27 μg/kg). Remaining compounds were below detection limits. Regarding co-occurrence (Figure 1A), CPT appeared alone in 26 samples and co-occurred with benzylisoquinoline alkaloids in 28 others. The most frequent multi-compound profile was the quaternary combination of CPT, protopine, allocryptopine, and berberine (*n* = 12).

### 3.3. Acute Toxicity of Identified Components

Zebrafish acute toxicity assays revealed distinct profiles for the five alkaloids (Figure 1B, Table S3). CPT exhibited the highest toxicity (LC_50_ = 0.047 μg/mL), approximately three orders of magnitude greater than the least toxic compound, berberine (LC_50_ = 167.10 μg/mL). The remaining compounds, protopine, veratramine, and allocryptopine, showed intermediate toxicity with LC_50_ values of 10.23, 20.72, and 30.54 μg/mL, respectively. CPT-exposed larvae exhibited pericardial and yolk sac edema, and spinal curvature (Figure 1C). Histopathological analysis revealed myocardial disorganization, reduced cell density, and hepatic vacuolation with nuclear pyknosis (Figure 1C), confirming CPT-induced cardiotoxicity and hepatotoxicity.

### 3.4. Acute Toxicity of Component Co-Exposure with Sugars

To evaluate interactions, larvae were exposed to CPT with various sugar matrices (Figure 1D, Table S3). Based on the Bliss independence model, a synergistic toxic effect between CPT and honey components was observed. Specifically, mimetic honey, glucose, and natural honey yielded SI values of 0.11, 0.13, and 0.57, respectively, indicating a significant potentiation of CPT toxicity. In contrast, fructose showed no such interaction (SI = 1.04), which is consistent with its negligible impact on the observed LC_50_ values.

**Figure 2:**
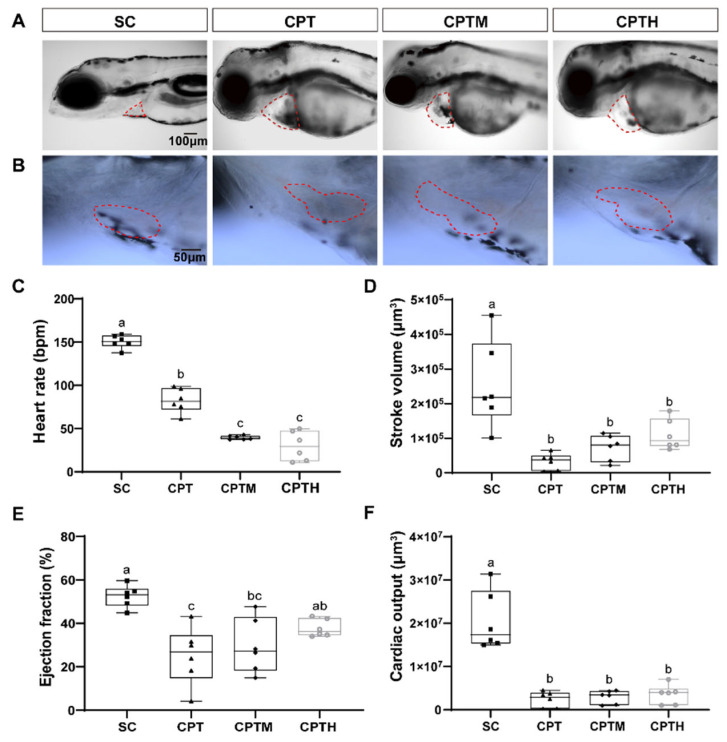
Effects of co-exposure of CPT with honey sugar matrix on cardiac morphology and function in zebrafish larvae. (**A**) Representative images of cardiac regions. (**B**) Magnified high-resolution images of the cardiac region. (**C**) Heart rate, (**D**) stroke volume, (**E**) ejection fraction, and (**F**) cardiac output. Data are presented as box plots (n = 6). Different letters denote a significant difference between groups (*p* < 0.05). Note: CPT, camptothecin; CPTM, CPT with mimetic honey; CPTH, CPT with honey. Pericardial area is outlined by a red dotted line.

Bright-field microscopy observations revealed consistent morphological abnormalities across co-exposure groups (Figure 2A,B). Marked pericardial edema and an elongated heart morphology were observed in groups exposed to CPT, CPTM, and CPTH. This unlooped morphology contrasts significantly with the normal looped structure observed in the SC, indicating compromised cardiac morphogenesis induced by the treatments. Importantly, the severity of cardiac malformations was most pronounced in the mimetic honey co-exposure group.

### 3.5. Cardiac Morpho-Functional Alterations After Co-Exposure

CPT treatment significantly impaired cardiac function (Figure 2C–F, Tables S8–S11), with HR, SV, EF, and CO decreasing by 45.2%, 87.4%, 52.1%, and 88.0% (*p* < 0.05), respectively, compared to the SC. In co-exposure groups, bradycardia was further exacerbated, with HR dropping to 39.60 bpm (CPTM) and 29.85 bpm (CPTH) (Figure 2C). Conversely, pumping efficiency (SV, EF, and CO) showed slight recovery in the CPTM group and more pronounced improvement in the CPTH group. In the CPTH group, EF returned to SC levels (a 50.1% increase relative to CPT), while SV increased 2.43-fold compared with CPT. However, due to severe bradycardia, the CO in the CPTH group remained 82.7% lower than the SC (*p* < 0.05).

### 3.6. Evaluation of Oxidative Stress After Co-Exposure

**Figure 3:**
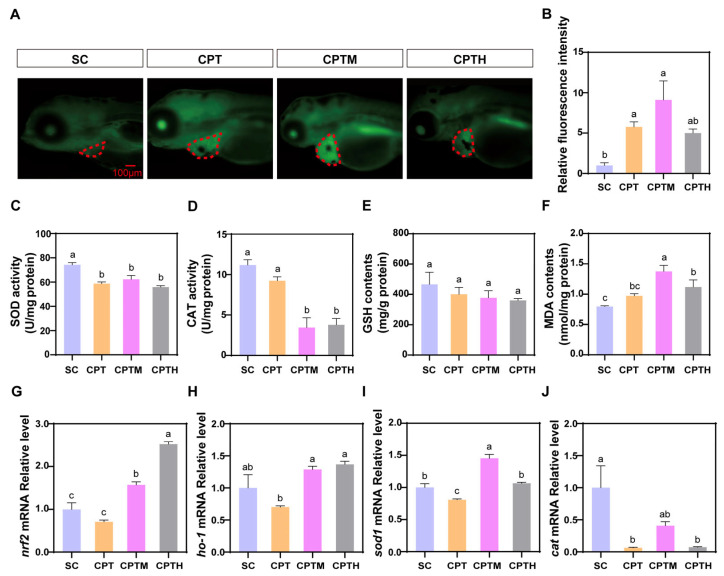
Effects of co-exposure of CPT with honey sugar matrix on oxidative stress in zebrafish larvae. (**A**) Representative fluorescence images of ROS production. (**B**) Quantification of cardiac relative fluorescence intensity (n = 10). (**C**) SOD activity, (**D**) CAT activity, (**E**) GSH levels, and (**F**) MDA levels (n = 3). (**G**–**J**) Relative mRNA expression of oxidative stress-related genes: *nrf2*, *ho-1*, *sod1*, and *cat* (n = 3). Different letters denote a significant difference between groups (*p* < 0.05). Note: CPT, camptothecin; CPTM, CPT with mimetic honey; CPTH, CPT with honey. Pericardial area is outlined by a red dotted line.

CPT treatment induced significant oxidative stress, characterized by a 4.77-fold increase in ROS levels and impaired antioxidant defenses, including reduced SOD and CAT activities and GSH content, leading to elevated MDA (Figure 3A–F, Tables S12–S16). These effects were exacerbated in the CPTM group, which exhibited the highest ROS and MDA levels, with CAT activity plummeting by 62.57% compared to the SC. Conversely, natural honey (CPTH) significantly mitigated this damage, showing lower ROS and MDA levels than CPTM (*p* < 0.05). At the transcriptional level, CPT downregulated *nrf2* and its targets (*ho-1*, *sod1*, *cat*) (Figure 3G–J, Table S17). Notably, both CPTM and CPTH induced a significant rebound in *nrf2*, *ho-1*, and *sod1* expression compared to CPT, with *nrf2* levels in the CPTH group being 60.89% higher than in CPTM.

### 3.7. Evaluation of Inflammatory Response After Co-Exposure

**Figure 4:**
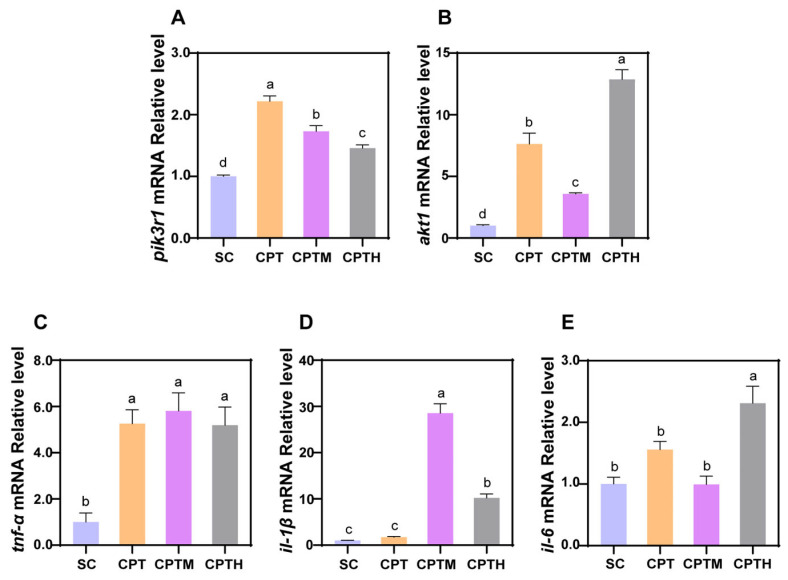
Effects of co-exposure of CPT with honey sugar matrix on PI3K/Akt pathway and inflammation in zebrafish larvae. (**A**,**B**) Relative mRNA levels of key genes in the PI3K/Akt signaling pathway (*pik3r1*, *akt1*) (n = 3). (**C**–**E**) Relative mRNA levels of pro-inflammatory cytokines (*tnf-α*, *il-1β*, and *il-6*) (n = 3). Different letters denote a significant difference between groups (*p* < 0.05).

To evaluate the inflammatory response, the mRNA expression levels of key signaling molecules and pro-inflammatory cytokines were analyzed (Figure 4, Table S17). Regarding the upstream signaling pathway, CPT treatment significantly upregulated the expression of *pik3r1* and *akt1* compared to the SC. In the CPTM group, these genes remained elevated relative to the SC but were significantly downregulated (22.0% and 53.1% respectively) compared to CPT alone. Notably, the CPTH group showed a further decrease in *pik3r1* but a 2.59-fold rebound in *akt1* relative to CPTM.

Regarding cytokines, *tnf-α* was uniformly upregulated (approximately 5-fold) across all treatments. While *il-1β* was unchanged by CPT alone, it was significantly upregulated in CPTM and CPTH groups; however, CPTH significantly attenuated *il-1β* expression (by 64.2%) relative to CPTM. Uniquely, *il-6* was only elevated in the CPTH group (2.31-fold higher than SC).

### 3.8. Evaluation of Apoptosis Induction After Co-Exposure

**Figure 5:**
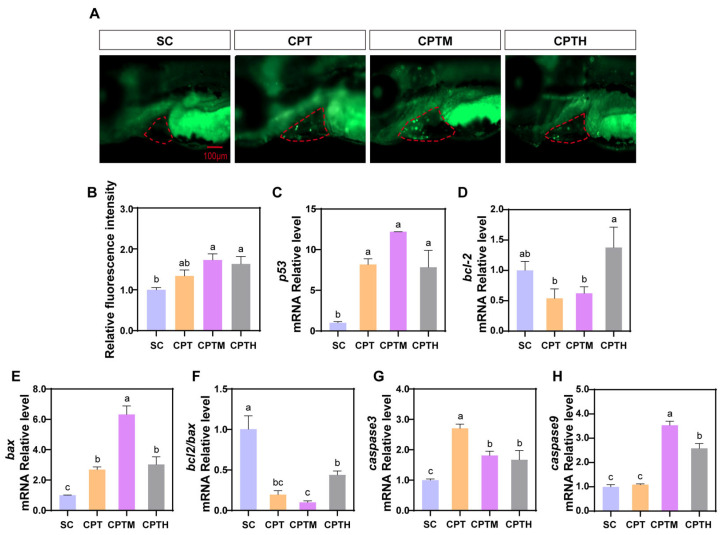
Apoptosis levels in zebrafish larvae at 5 dpf after exposure to CPT with honey sugar matrix. (**A**) Representative fluorescence images of zebrafish larvae stained with AO. (**B**) Quantification of apoptotic cells relative fluorescence intensity (n = 10). (**C**–**H**) Expression of apoptosis-related genes (*p53*, *bcl-2*, *bax*, *caspase3*, and *caspase9*) and the calculated *bcl-2*/*bax* ratio (n = 3). Different letters denote a significant difference between groups (*p* < 0.05). Note: CPT, camptothecin; CPTM, CPT with mimetic honey; CPTH, CPT with honey. Pericardial area is outlined by a red dotted line.

AO staining showed that all treatments induced significant cardiac apoptosis compared to the SC, with the CPTM group exhibiting the most pronounced effect, a 1.73-fold increase (*p* < 0.05; Figure 5A,B). At the transcriptional level, CPT significantly upregulated pro-apoptotic *p53* and *bax* while downregulating anti-apoptotic *bcl2*, leading to increased *caspase-3* expression (Figure 5C–H). These changes were further exacerbated in the CPTM group, where the *bcl2/bax* ratio was suppressed by 48.91% and *caspase-9* was uniquely upregulated 2.24-fold compared to CPT alone. In the CPTH group, although *bcl2* was partially recovered, the *bcl2/bax* ratio and levels of *p53*, *caspase-3*, and *caspase-9* remained similar to the CPTM group, indicating a sustained apoptotic cascade.

## 4. Discussion

Phytotoxins derived from specific nectariferous plants can be transferred to honey, potentially posing significant health risks to consumers. To date, only a limited number of such phytotoxic constituents have been identified, primarily including triptolide [5,13], tutin [14], grayanotoxins [15], gelsedine-type alkaloids [16,17]. The potential presence of unidentified phytotoxins in honey has become a growing concern, as an important scientific gap in honey safety lies not only in the insufficient understanding of unknown toxic components but also in the unclear mechanisms by which the intrinsic sugar matrix modulates toxicity.

In this study, a sensitive multi-marker analytical method was developed and fully validated by optimized LC-MS/MS conditions, enabling the accurate quantification of toxic alkaloids with reported low median lethal dose (LD_50_). Analysis of 150 *Apis cerana* honey samples using this established platform successfully identified unique alkaloid profile characteristics [18,19]. The analysis revealed a high prevalence of CPT in honey samples, a finding closely linked to the extensive distribution of *Camptotheca acuminata* and *Nothapodytes pittosporoides* across the Yangtze River Basin and southern China [20,21]. This reflects regional contamination patterns similar to those observed in New Zealand honey, where tutin levels are driven by local toxic flora [22]. Furthermore, the co-occurrence of benzylisoquinoline alkaloids (protopine, allocryptopine, and berberine) in samples collected near *Macleaya cordata* plantations provides direct evidence of toxin transfer from medicinal crops to honey. Collectively, these findings underscore the profound impact of local botanical distribution and bee foraging behavior on the chemical safety of honey.

Acute toxicity assays revealed distinct risk profiles among the detected alkaloids, with CPT emerging as the most potent toxin in the zebrafish model. Furthermore, the results highlighted significant interspecies sensitivity variations. For instance, while veratramine is highly lethal to mice (LD_50_ = 15.9 mg/kg) [23], it manifested only moderate toxicity in zebrafish (LC_50_ = 20.72 μg/mL). This discrepancy is likely attributable to toxicokinetic differences between mammals and teleosts, suggesting that species-specific biological responses must be integrated into honey safety assessments.

In stark contrast, CPT stands out for its potent lethality across multiple species. Functioning as a Topoisomerase I (Topo I) inhibitor, CPT induces DNA double-strand breaks, a mechanism particularly devastating to the rapidly proliferating cells of zebrafish larvae [24]. Our results confirm that CPT exerts specific and severe cardiotoxicity, manifested as pericardial edema, myocardial disorganization, and reduced cardiomyocyte density. This finding is consistent with a study by Song et al., who found that CPT demonstrated the potential to cause cardiovascular dysfunction [24]. Given this intrinsic potency, determining whether the honey sugar matrix alters CPT’s toxicological behavior becomes a critical safety question.

In zebrafish models, exposure to CPT with honey sugar matrices induced severe bradycardia (Figure 2C), in contrast to reports by Fakhlaei that sugar-adulterated honey induces tachycardia in zebrafish embryos [25]. This suggests that CPT’s potent inhibitory effect on HR dominates the physiological response, while the sugar matrix may exacerbate its cardiotoxicity through synergistic effects. Despite markedly reduced heart rates, EF in CPTH and CPTM groups exhibited a trend toward relative maintenance or recovery (Figure 2E), potentially reflecting an intrinsic compensatory mechanism that maintains CO at low heart rates by enhancing myocardial contractility [26]. However, none of the treatment groups ultimately restored CO to normal levels (Figure 2F), highlighting systemic cardiac dysfunction. This pattern of heart failure is consistent with the findings of Wang et al., who demonstrated that plant alkaloids such as sanguinarine induce myocardial injury where compensatory mechanisms are insufficient to counteract the resulting pathological damage [27].

Studies have revealed that exposure to CPT causes an imbalance in redox reactions within myocardial cells, leading to excessive ROS production [28]. As a primary defense, SOD eliminates superoxides, while co-exposure with the mimetic honey significantly exacerbated oxidative damage, disrupting the equilibrium between ROS generation and clearance. This intensified oxidative stress resulted in a sharp rise in MDA levels, indicating severe lipid peroxidation. Such exacerbation is likely attributable to sugar-induced enzyme inactivation and mitochondrial dysfunction. Consistent with the findings of Mofidi Najjar et al. [29], high glucose levels can induce non-enzymatic glycation of CAT, structurally impairing its enzymatic activity. Furthermore, aligning with the research by Gugliucci on fructose-mediated damage, the high reactivity of fructose promotes mitochondrial membrane permeability and subsequent superoxide leakage [30]. Consequently, the precipitous decline in CAT activity and the surge in ROS in the CPTM group underscore the synergistic toxicity of the sugar matrix, which compromises both antioxidant enzyme integrity and mitochondrial stability.

In the present study, the partial recovery of *nrf2*, *ho-1*, and *sod1* in the CPTM and CPTH groups suggests that co-exposure to CPT with sugars may mitigate oxidative damage by reactivating the Nrf2 signaling pathway. These findings regarding natural honey in the CPTH group are consistent with historical records documenting its ability to reduce toxicity and enhance medicinal efficacy [31]. This mitigating effect is likely attributed to the synergistic interplay of bioactive non-sugar constituents, particularly polyphenols, which help to counterbalance the pro-oxidant environment [32,33]. As noted by Ranneh et al. [34], the diverse range of polyphenolic compounds found in honey, such as gallic acid and protocatechuic acid, serves as the fundamental basis for its potent antioxidant and anti-inflammatory biological activities.

In our study, the significant upregulation of *akt1* mRNA levels suggests that the PI3K/Akt signaling pathway is highly activated in this stress response [35]. As a central regulatory hub of the inflammatory response, the NF-κB pathway is one of the known key downstream effectors of the PI3K/Akt pathway [36,37]. Thus, our findings support a model of molecular mechanism: the CPT enhances NF-κB activity through the PI3K/Akt signaling axis, thereby initiating a pro-inflammatory cascade response. This finding is consistent with the seminal study by Huang et al., which showed that CPT-induced DNA damage can activate NF-κB [37]. Notably, the sustained activation of NF-κB has a dual effect: under cumulative toxic exposure, it is dysregulated from a protective response to a key driver of chronic inflammation and tissue damage [38]. Studies have shown that AGEs, formed from sugar components might activate the AGE-RAGE/NF-κB signaling axis [35]. This activation synergistically amplifies NF-κB signaling and drives the over-expression of pro-inflammatory cytokines, such as *il*-*1β* and *tnf-α*. This potential mechanism aligns closely with the observed phenomenon where mimetic honey exacerbates inflammation and cardiotoxicity induced by CPT. This suggests that AGEs may play a mediating role in this process.

As a core regulator of cellular stress, the activation of *p53* typically initiates the intrinsic apoptotic cascade [39]. Previous studies indicate that *p53* converts cellular stress signals into pro-apoptotic stimuli by upregulating pro-apoptotic factors such as Bax while simultaneously suppressing anti-apoptotic proteins like Bcl-2 [36]. This mechanism aligns closely with the gene expression trends observed at the transcriptional level in our study. Consistent with prior research on fructose-induced apoptosis in L6 cells, an imbalance in the Bcl-2/Bax ratio leads to the loss of mitochondrial membrane potential and increased permeability [40]. In the present study, our results demonstrated a significant decrease in the *bcl-2*/*bax* ratio in the CPTM group. Consistent with prior research on fructose-mediated damage [41], such an imbalance often leads to the loss of mitochondrial membrane potential and increased permeability. Notably, this pro-apoptotic trend was mitigated in the CPTH group, aligning with the findings of Farkhondeh et al. [42], who emphasized that chrysin, as a key flavonoid in honey, can preserve mitochondrial integrity by downregulating *bax* expression and upregulating *bcl-2* levels.

## 5. Conclusions

This study systematically identified phytotoxins in raw honey from southern-central China, highlighting CPT as a primary contaminant due to its high prevalence and potent toxicity. Our findings demonstrate that the intrinsic sugar matrix significantly exacerbates CPT-induced cardiotoxicity through an integrated oxidative–inflammatory–apoptotic cascade. In contrast, honey’s non-sugar bioactive components were found to provide partial antagonistic protection against this toxicity. However, as this screening focused on 29 specific toxins within a defined geographic scope, further research is needed to validate these risks in honey from broader origins. Future studies should also isolate the specific non-sugar compounds responsible for the observed mitigation. Overall, these insights underscore a previously overlooked matrix-dependent risk, emphasizing the necessity for comprehensive safety assessments that account for the synergistic effects of honey components on contaminants.

## Data Availability

The original contributions presented in this study are included in the article/Supplementary Materials. Further inquiries can be directed to the corresponding authors.

## Supplementary Materials

The following supporting information can be downloaded at https://www.mdpi.com/article/10.3390/foods15061058/s1, Table S1. Origin information of raw honey samples. Table S2. Parameters for quantitative analysis of the analytes in LC-QTRAP MS/MS MRM mode. Table S3. Concentrations and LC50 values of acute toxicity test of on zebrafish. Table S4. Primer list. Table S5. Summary of method performance of the analytes in raw honey using LC-QTRAP MS/MS. Table S6. Recoveries of method performance of the analytes in honey using LC-QTRAP MS/MS. Table S7. Contents of target compounds in raw honey samples. Table S8. Heart rate (bpm) across experimental groups. Table S9. Stroke volume (μm3) across experimental groups. Table S10. Ejection fraction (%) across experimental groups. Table S11. Cardiac output (μm^3^) across experimental groups. Table S12. Relative fluorescence intensity of ROS across experimental groups. Table S13. SOD activity (U/mgprot) across experimental groups. Table S14. CAT activity (U/mgprot) across experimental groups. Table S15. GSH contents (mg/gprot) across experimental groups. Table S16. MDA contents (nmol/mgprot) across experimental groups. Table S17. Original data of RT-PCR (caspase9) across experimental groups. Table S18. Relative fluorescence intensity of AO across experimental groups. Text S1. Supplementary Methods, including LC-MS/MS quantitation methods, chemicals and cautions, and LC-MS/MS MRM parameters.

## Abbreviations

AO, acridine orange; CO, cardiac output; CPT, camptothecin; CPTH, camptothecin with honey; CPTM, camptothecin with mimetic honey; EF, ejection fraction; HR, heart rate; LC_50_, median lethal concentration; SC, solvent control; SV, stroke volume.
