# Supplementary material for: Screening of Phytotoxins in Raw Honey and the Honey Sugar Matrix’s Modulatory Effects on Their Toxicity
Source: Foods. 2026 Mar 17;15(6):1058. doi: 10.3390/foods15061058 (PMC13025968; doi:10.3390/foods15061058)
Supplement: Supplementary file 1 [file foods-15-01058-s001.zip › Supplementary Methods_Text S1.pdf]

Text S1. Supplementary Methods

LC-MS/MS quantitative analysis

An LC-MS/MS method was developed for the quantification of target analytes using a Shimadzu UFLC system (CBM-30A controller, Shimadzu, Japan) coupled to an AB SCIEX 4500 QTRAP mass spectrometer (Applied Biosystems, USA). Chromatographic separation was conducted on a Waters ACQUITY UPLC HSS T3 C18 column (100 × 2.1 mm, 1.8 μm, Waters, USA) at a temperature of 40 ℃. Formic acid (0.1%, v/v) in water and acetonitrile served as mobile phases A and B, respectively. The gradient program for mobile phase A (with a flow rate of 0.35 mL/min) was as follows: maintained at 95% for 1 minute; linearly decreased to 5% over the subsequent 11 minutes; held at 5% for 1 minute before a rapid increment back to 95%; and finally, equilibrated for 5 minutes with an initial proportion of 95% mobile phase A. The injection volume was 5 μL, and the autosampler was maintained at 4 ℃.

The mass spectrometer was operated using an electrospray ionization (ESI) source in positive mode. Multiple reaction monitoring (MRM) scans were performed for the detection of target compounds under the following conditions: ion source voltage, 5500 V; curtain gas (nitrogen), 35 psi; dry gas, 60 psi; ion source temperature, 550 °C; collision induced ionization, 2/-2; The declustering potentials (DP) and collision energy (CE) values were optimized via the automatic function of Analyst software, and the comprehensive MRM transition parameters were provided in Table S2.

Method performance and quantitation

The matrix effects (MEs) of 17 analytes were measured by diluting a mixed standard solution (2 $\mu g \cdot mL^{-1}$) to a final concentration of 10 $ng \cdot mL^{-1}$ and 1 $ng \cdot mL^{-1}$ in methanol and a mimetic honey matrix, respectively. The matrix factor for each analyte was calculated by ascertaining the ratio of the peak area in the presence of the matrix to that in methanol. The linearity was examined via sequential spiking of the mixed standard solution (2 $\mu g \cdot mL^{-1}$) into matrix extracts of mimetic honey as a diluent to achieve a concentration range of 0.01~500 $ng \cdot mL^{-1}$. The limits of quantitation (LOQs) were determined using the signal-to-noise (S/N) approach, with an S/N ratio greater than 10 for the LOQs. Precision was measured by six consecutive injections of a 20 $ng \cdot mL^{-1}$ standard solution, repeatability was assessed from six parallel determinations, and stability was evaluated by detections at 0 h, 4 h, 8 h, 12 h, 16 h, 20 h, and 24h, all of these evaluations were quantified using the relative standard deviations (RSDs) of the peak areas of these analytes. Recoveries were evaluated through the external standard method by spiking three distinct concentrations (low, medium, high) of the mixed standard solutions into five replicates of the mimetic honey samples, followed by the calculation based on measured and theoretical concentrations of spiked mixed standards.

Chemicals and caution

Dimethyl Sulfoxide is carcinogenic in laboratory animals. They should be handled with extreme care, using proper personal protective equipment and a well-ventilated hood. Chemicals used in this study, unless indicated otherwise, were purchased from Sinopharm Chemical Reagent Co., Ltd. (China).
